# Comparison of quadricep motor evoked potentials between different surgical positions during total hip arthroplasty

**DOI:** 10.1007/s10877-025-01308-9

**Published:** 2025-06-10

**Authors:** Wataru Shirahata, Ryohei Takada, Naoto Watanabe, Kazumasa Miyatake, Ayami Sato, Kanako Minegishi, Toshitaka Yoshii, Hideyuki Koga

**Affiliations:** Department of Joint Surgery and Sports Medicine, Institute of Science Tokyo, 1-5-45, Yushima, Bunkyo-ku, Tokyo, 113-8510 Japan

**Keywords:** Femoral nerve palsy, Total hip arthroplasty, Intraoperative position, Anterolateral approach, Transcranial electrical stimulation motor-evoked potential

## Abstract

The influence of intraoperative position on femoral nerve palsy after total hip arthroplasty (THA) remains unclear. Therefore, we evaluated the effect of intraoperative position on quadricep motor-evoked potential monitoring in patients undergoing THA using an anterolateral approach. We included patients who underwent primary THA using the anterolateral approach at our hospital between June 2021 and January 2024 with available data on intraoperative quadricep using transcranial electrical stimulation motor-evoked potential. Patient characteristics were compared between the supine and lateral position groups. Intraoperative quadricep MEP were evaluated at the beginning of surgery, after anterior acetabular retractor placement, after acetabular retractor placement, and before wound closure. The MEP amplitude at surgery start was set to 100%, and the change in amplitude at each time point was compared between positions. Ten patients were placed in the supine and lateral positions. Patient background did not differ significantly between the groups, and no postoperative paralysis was observed. The residual rates of quadriceps MEP were significantly lower in the supine position than the lateral position at all three time points (*p* < 0.05). Intraoperative quadricep motor-evoked potential monitoring in primary THA using the anterolateral approach showed significantly lower MEP amplitude in the supine position than in the lateral position at all three time points. Therefore, the lateral position may decrease femoral nerve palsy risk after THA.

## Introduction

Postoperative nerve palsy is a serious complication of total hip arthroplasty (THA) that significantly impacts patient functional prognosis. The reported incidence rates of nerve palsy for primary and revision THA are 0.1–3.7% and 0–7.6%, respectively [1–3]. The risk factors for nerve palsy after THA include young age, smoking, postoperative lumbar spine surgery, and late operative start time [4]. Among nerve palsies, the reported incidence of femoral nerve palsy, which often occurs due to inappropriate anterior acetabular retractor placement, is 0.1–2.4% [5, 6]. The distance between the femoral nerve and the anterior acetabular edge (dFN) is positively correlated with patient height, and a shorter dFN is associated with a higher risk of developing femoral nerve palsy after THA [7]. Significantly shorter stature and dFN in the femoral nerve palsy group than in the non-paralyzed group has been reported [8]. In addition, the dFN was significantly longer in the lateral position than in the supine position [9].

Although the results of these studies suggest that the risk of femoral nerve palsy is lower in lateral-position surgery, to our knowledge, no studies have investigated the differences in the reduction of potentials according to the intraoperative position.

Therefore, we hypothesized that femoral nerve-mediated MEP during THA via the anterolateral approach would be lower in the supine position than in the lateral position. This study aimed to evaluate this hypothesis and determine the effect of the intraoperative position on quadricep MEP during THA.

## Materials and methods

This study adhered to the principles of the Declaration of Helsinki and was approved by the Research Ethics Committee of Institute of Science Tokyo (No. M2000-1099, Postoperative Clinical Study of Total Hip Replacement Surgery). All patients included in this study provided written informed consent before their participation and surgery.

Patients.

This study was conducted at a single university hospital and included patients undergoing THA with an anterolateral approach who underwent motor-evoked potential (MEP) monitoring at the surgeon’s discretion between June 2021 and January 2024. Patients who underwent leg extension of > 2 cm or Crowe classification Type III or higher were excluded.

Transcranial electrical stimulation motor-evoked potentials (Tc-MEPs).

**Fig. 1:**
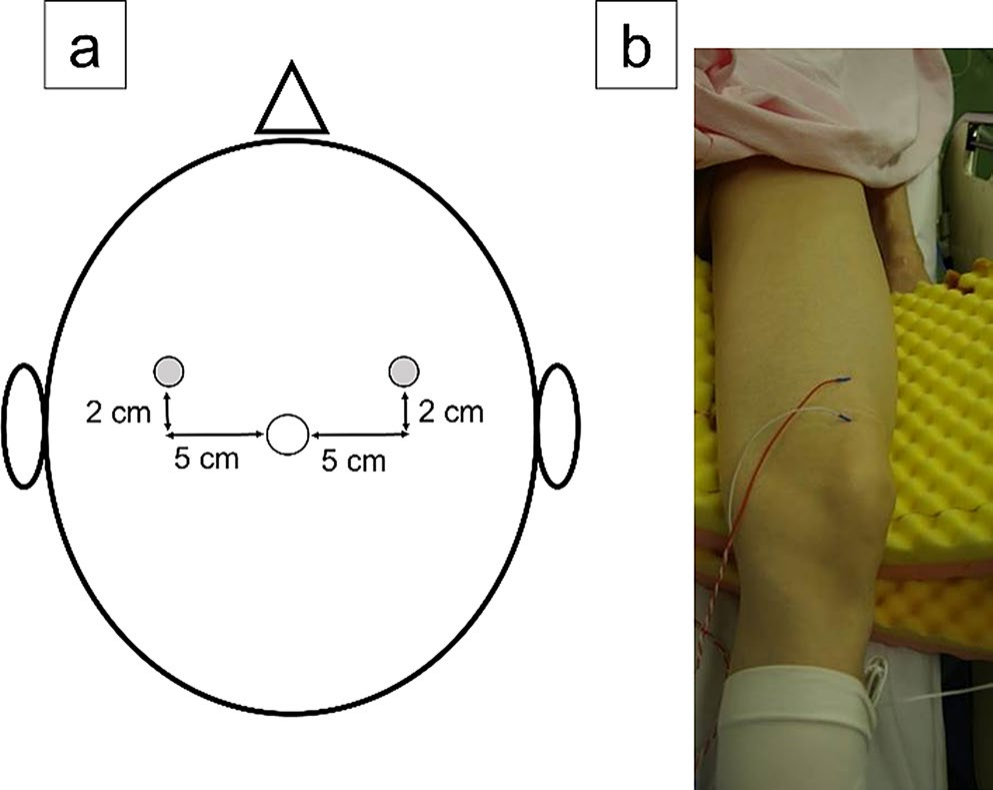
Electrode locations on the head (**a**) and lower extremity (**b**). (**a**) Corkscrew head-stimulating electrodes located 5 cm lateral and 2 cm anterior to the vertex. (**b**) Needle subcutaneous electrodes inserted into the belly of the anteromedial side of the distal quadriceps muscle

Tc-MEPs were evaluated using the muscle potentials of quadriceps. Corkscrew electrodes were placed at two points on either side of the head, 2 cm anterior and 5 cm lateral to the vertex (Fig. 1a). A train of five stimulus pulses with an inter-stimulus interval (ISI) of 2.0 ms was applied with a pulse width of 0.5 ms and current stimulation at a stimulus intensity of 200 mA. The recording electrode was a disposable subcutaneous needle implanted into the quadriceps muscle (Fig. 1b). The quadriceps muscle potentials were measured at four time points: at the beginning of surgery, after anterior acetabular retractor placement, after placement of the four acetabular retractors, and before wound closure.

### Anesthesia

All surgeries were performed under general anesthesia. All patients were anesthetized using total intravenous anesthesia to prevent the effects of inhaled anesthetics on reduced muscle potential. An initial dose of 0.6–0.7 mg/kg of rocuronium was administered, and supplemental doses were provided as necessary in cases of difficult intubation. Additionally, sugammadex was administered at a dose of 200 mg as a standard protocol before potentiometry to antagonize the muscle relaxant effect. Surgical procedures and measurement of MEP commenced following confirmation by the anesthesiologist that neuromuscular blockade had been sufficiently reversed, as assessed by the train-of-four (TOF) value of the ulnar nerve at the wrist.

### Surgical procedure

**Fig. 2:**
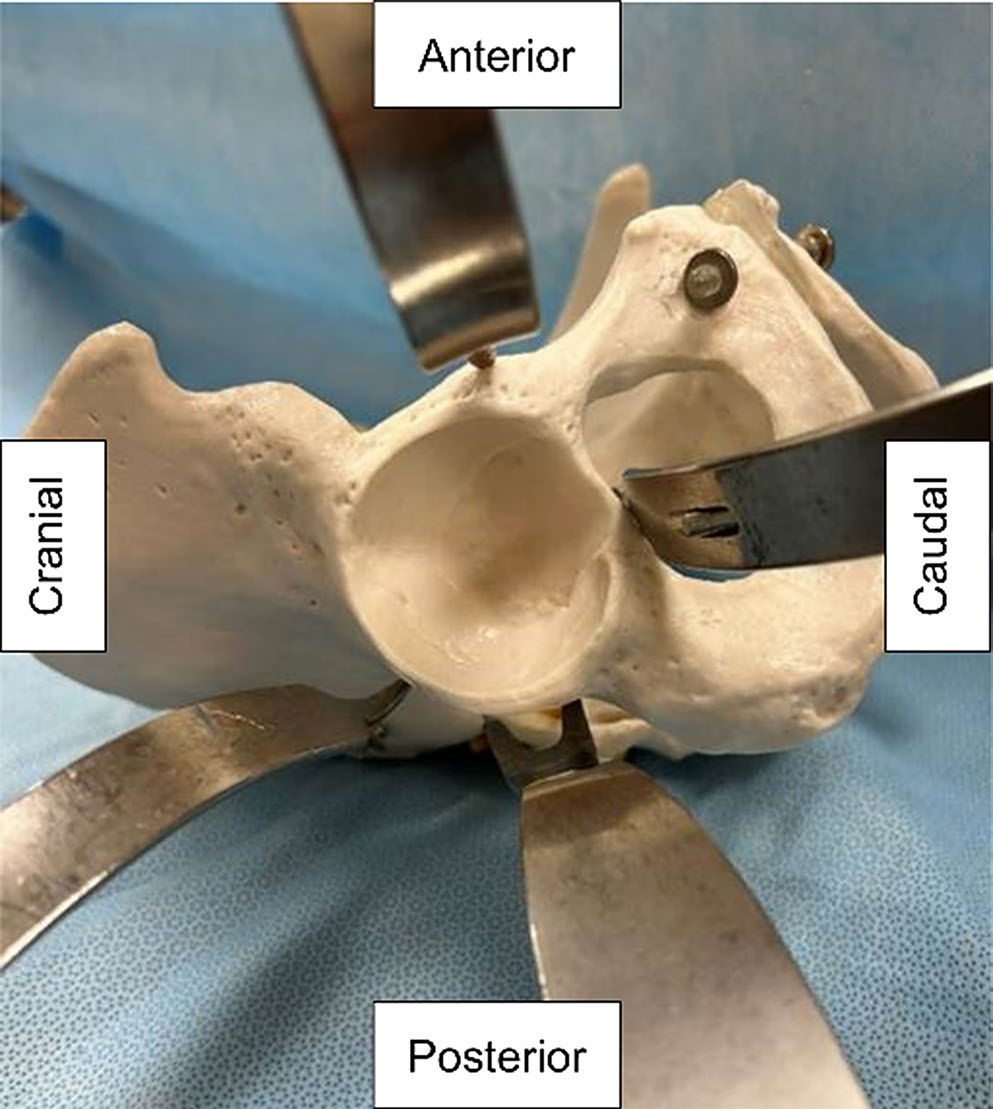
Positioning of the acetabular retractors. Model showing the position of the acetabular retractors on the right hip joint during total hip arthroplasty. Double- and single-prong retractors are used in the posterior and other regions, respectively

No specific criteria were applied to indicate the choice of supine or lateral position. Three surgeons performed the procedures. A modified Watson–Jones approach was used in the supine and lateral positions. A 10–12-cm skin incision was made on the anterolateral side of the hip joint and expanded between the tensor fascia latae and gluteus medius. After arthrotomy, osteotomy of the femoral neck was performed, and an anterior acetabular retractor was placed between the iliopsoas muscle and the acetabulum during acetabular manipulation in both supine and lateral positions. Additional retractors were then placed on the cranial, caudal, and posterior sides of the acetabulum, for a total of four retractors (Fig. 2). The same set of retractors was used in all cases. No traction table was used. Cementless cups were used in all cases.

### Evaluation items

**Fig. 3:**
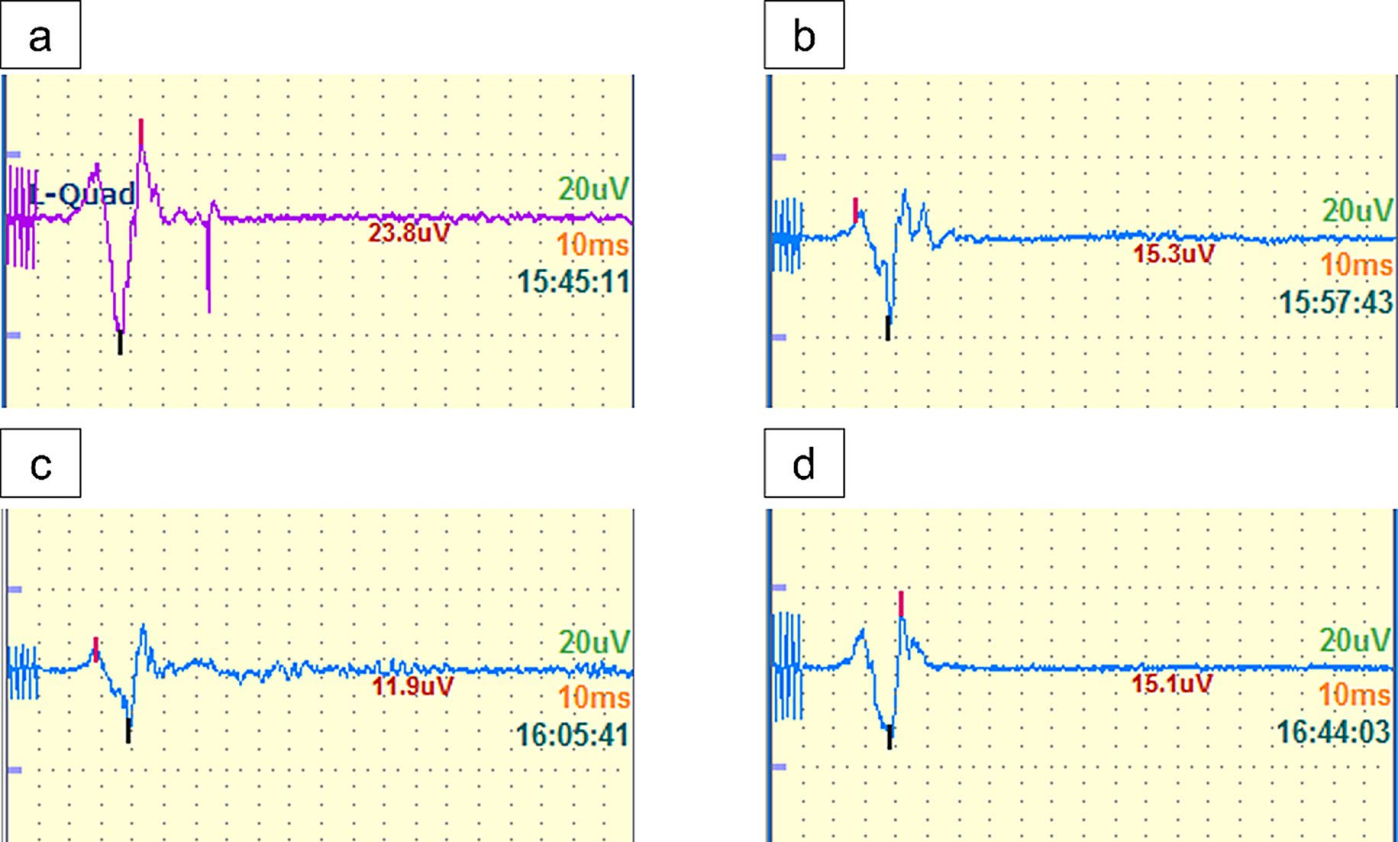
Waveform and amplitude of the MEP potential. The intraoperative monitoring screen used for recording. Typical waveform of motor-evoked potential (MEP) in the quadriceps muscle. **a**: Waveform at the beginning of surgery (baseline). **b**: Waveform after placement of the anterior acetabular retractor. **c**: Waveform after placement of the four acetabular retractors. **d**: Waveform before wound closure

Patient background, age, sex, height, weight, and body mass index (BMI); operative time; blood loss; dFN; Japanese Orthopaedic Association score [10]; diagnosis; left/right; Crowe classification; and intraoperative quadriceps muscle MEP amplitudes were investigated. The amplitude at the beginning of surgery was defined as the baseline (100%) (Fig. 3), and the residual rates after potential reduction at three points during the placement of the anterior acetabular retractor, four acetabular retractors, and before wound closure were compared between the supine and lateral positions. The presence of postoperative nerve palsy was also assessed. In addition, the correlation between the dFN and the residual rate of the MEP amplitude was evaluated at each time point.

**Fig. 4:**
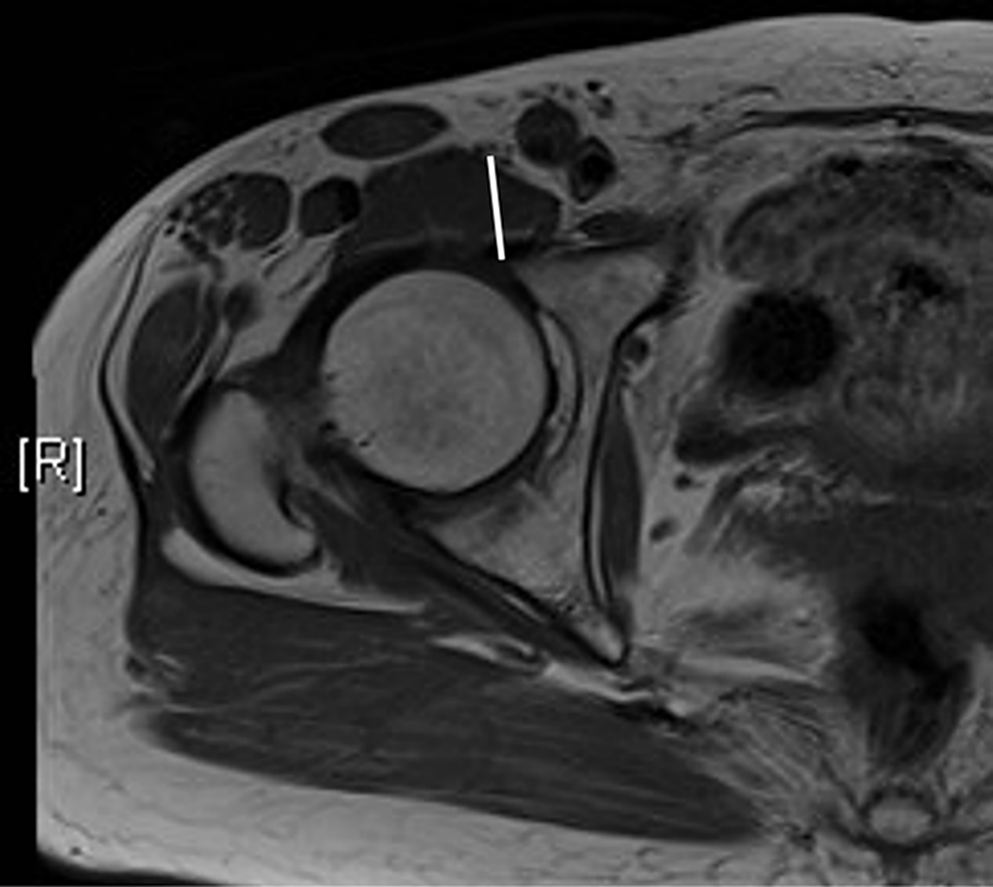
MRI measurement of the Dfn. Axial T1–weighted MRI scan of the right hip at the level of the center of the femoral head in a 67-year-old woman. MRI, magnetic resonance imaging; white line: dFN. dFN, distance between the anterior acetabular edge and femoral nerve

The dFN levels were measured using axial magnetic resonance imaging. The distance between the anterior acetabular edge and femoral nerve was measured at the level of the hip center using the digital caliper tool in INFINITT’s Picture Archiving and Communications System (Fig. 4).

### Statistical analysis

We used an unpaired t-test to analyze each continuous variable related to patient background, and a χ2 test for the left and right sides and the Crowe classification. For the percentage of MEP amplitude reduction, we used the Mann–Whitney U test. For correlation analysis, we used the Pearson correlation coefficient. GraphPad Prism 7 (GraphPad Software, La Jolla, CA, USA) was used for all statistical analyses. Power analysis was conducted using G*Power software, version 3.1.9.7 (Heinrich-Heine-Universität Düsseldorf, Düsseldorf, Germany), to estimate the number of patients required to detect a statistically significant difference. The effect sizes were determined from data in previous literature and calculated with Cohen’s d = 1.2, α = 0.05, and 1-β = 0.8. The results indicated that 20 patients were required. Statistical significance was set at *p* < 0.05.

## Results

We recorded intraoperative quadricep MEP during primary THA in 32 hips during the study period; among these, six with leg extension > 2 cm, four with missing data, and two with Crowe classification type 3 were excluded from the analysis. Intraoperative quadriceps potentials were evaluated in 10 patients each in the supine and lateral positions. The patients’ backgrounds did not differ significantly between the groups (Table 1). No cases of postoperative femoral nerve palsy were observed.

**Fig. 5:**
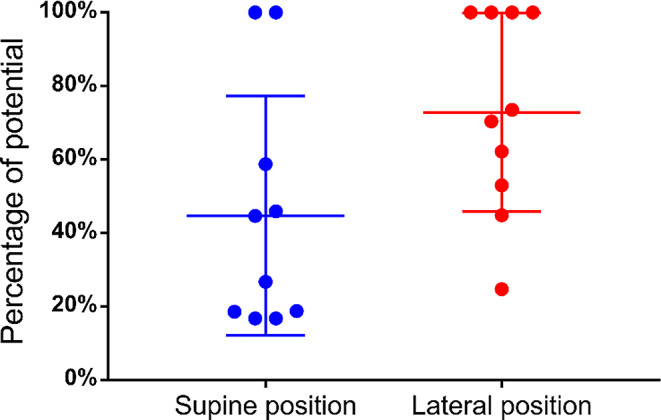
Changes in potential after anterior acetabular retractor placement. ●: Residual potential after anterior acetabular retractor placement compared with the baseline measured at surgery initiation. Long lines: mean percentage; short lines: interquartile ranges. The reduction is significantly larger in the supine group (44.7% [16.8–100%] vs. 72.9% [24.7–100%])

**Fig. 6:**
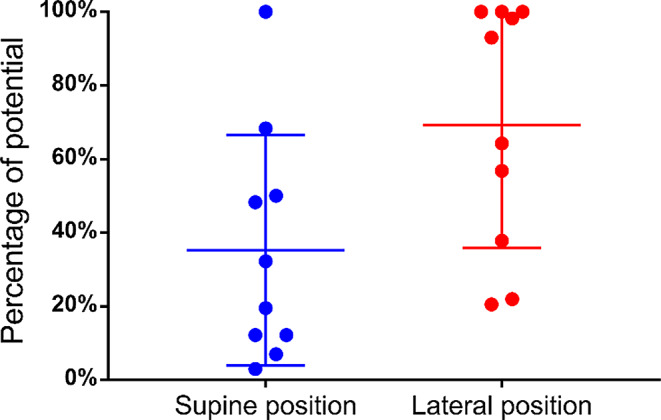
Changes in potential after placement of the four acetabular retractors. ●: Residual potentials after acetabular retractor placement compared with the baseline values measured at surgery initiation. Long lines: mean percentage; short lines: interquartile ranges. The potential reduction is significantly larger in the supine group (35.3% [3.0–100%] vs. 69.3% [20.5–100%]

**Fig. 7:**
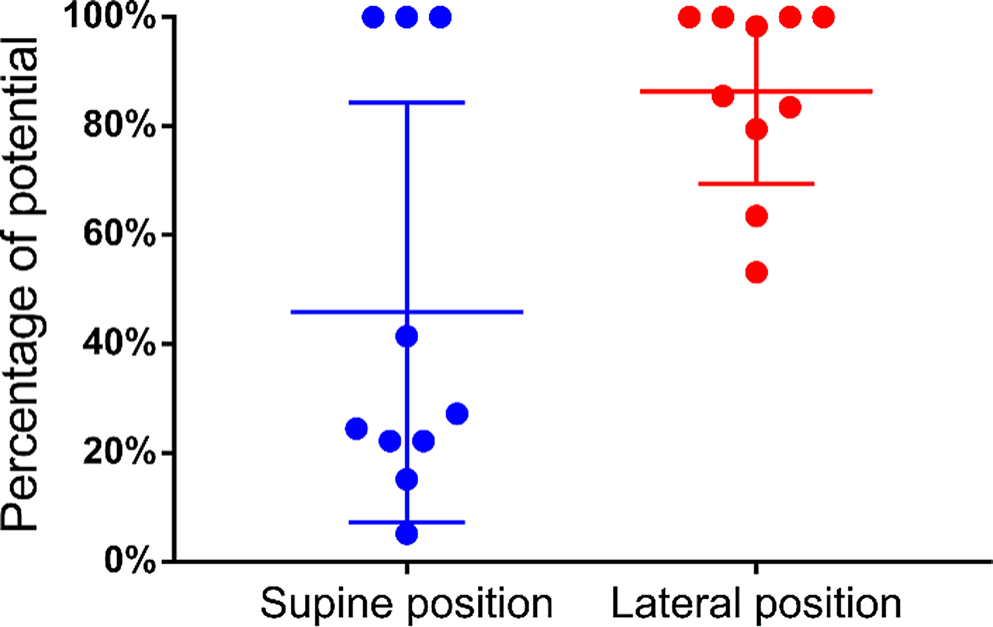
Changes in potential before wound closure. ●: Residual potentials before wound closure compared with the baseline values measured at surgery initiation. Long lines: mean percentage; short lines: interquartile ranges. The potential reduction was significantly greater in the supine group (45.8% [5.2–100%] vs. 86.3% [53.1–100%])

After placement of the anterior acetabular retractor, we observed potential reduction in both groups, with residual potentials of 44.7% (16.8–100%) and 72.9% (24.7–100%) in the supine and lateral position groups, respectively (Fig. 5). The potential decrease in the supine position group was significant compared with that in the lateral position group (*p* = 0.02). The potential decrease values in the lateral and supine position groups were 35.3% (3–100%) and 69.3% (20.5–100%), respectively, for the placement of the four acetabular retractors (*p* = 0.015) (Fig. 6) and 45.8 (5.2–100%) and 86.3 (53.1–100%), respectively, before wound closure (*p* = 0.023), with significant potential reduction in the supine group (Fig. 7).

**Fig. 8:**
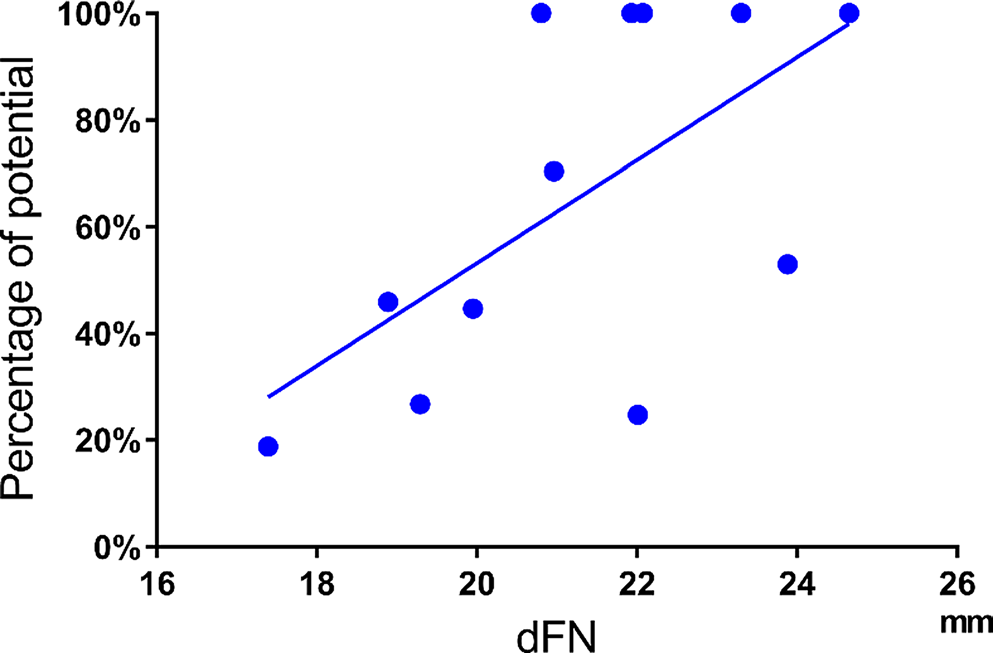
Correlation between dFN and potential after anterior acetabular retractor placement. ●: Residual rates of the potentials after anterior acetabular retractor placement relative to the preoperative potential amplitude. Solid line: approximate straight line. Correlation coefficient: 0.62 (*p* = 0.03, moderate positive). dFN, distance between the femoral nerve and the anterior acetabular edge

**Fig. 9:**
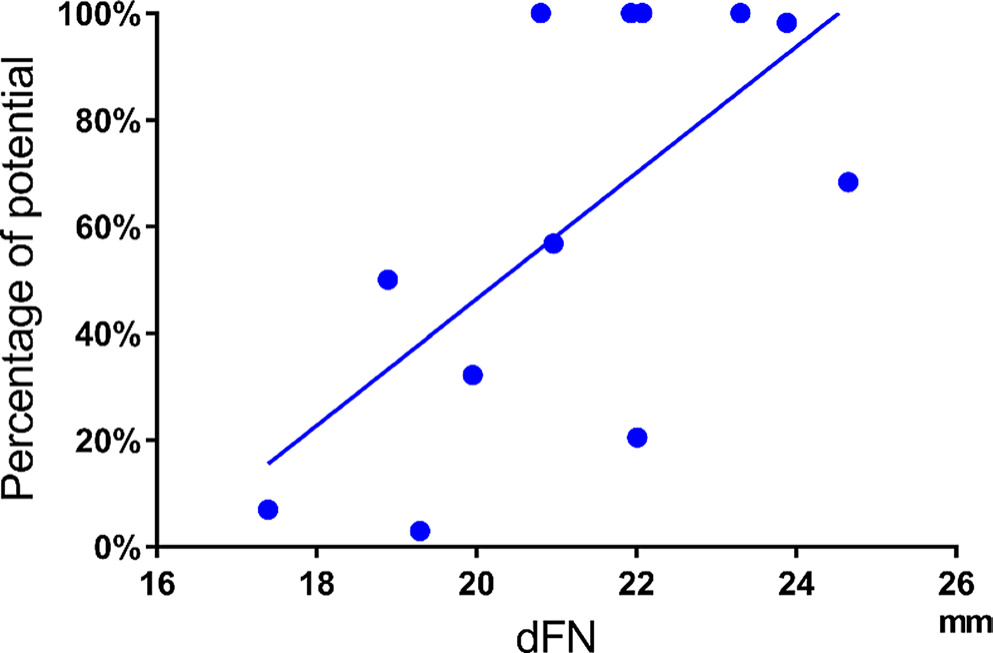
Correlation between dFN and potential after placement of four acetabular retractors. ●: Residual rates of the potentials after placement of all acetabular retractors relative to the preoperative potential amplitude. Solid line: approximate straight line

**Fig. 10:**
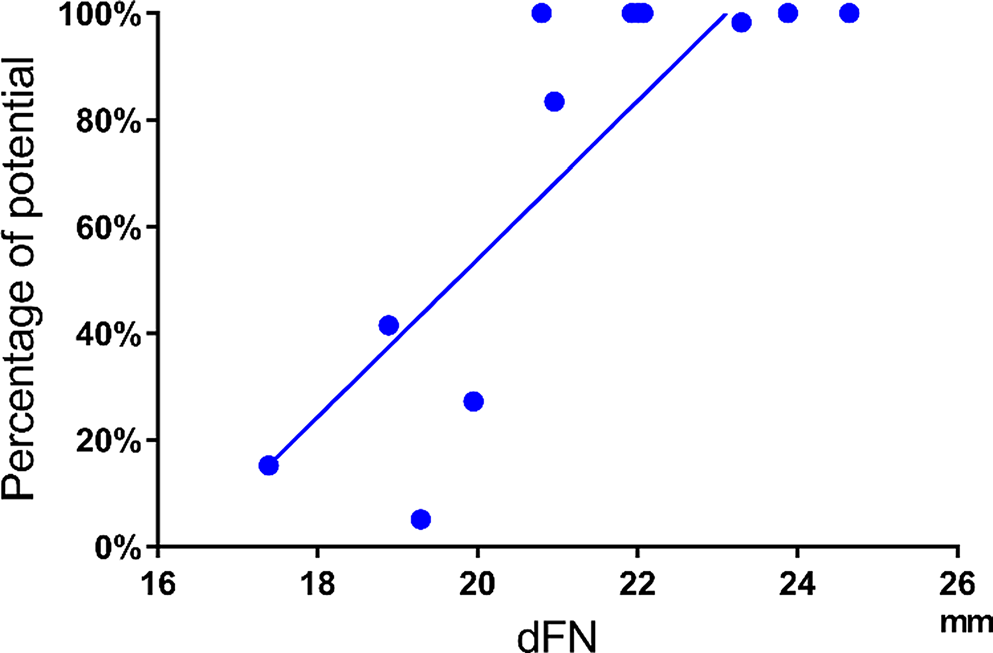
Correlations between dFN and potential before wound closure. ●: Residual rates of the potentials before wound closure relative to the preoperative potential amplitude. Solid line: approximate straight line. Correlation coefficient: 0.83 (*p* = 0.001, strong positive). dFN, distance between the femoral nerve and the anterior acetabular edge

The Pearson correlation coefficients between the dFN and the residual rate of electrical potential were 0.62 (*p* = 0.03) after anterior acetabular retractor placement (Fig. 8), 0.66 (*p* = 0.02) after placement of the four acetabular retractors (Fig. 9), and 0.83 (*p* = 0.001) before wound closure, showing significant positive correlations (Fig. 10).

**Table 1:** Participant demographic and clinical characteristics

	Supine position *N* = 10	Lateral position *N* = 10	*P*-value
Age Sex (female, %) Height (cm) Weight (kg) BMI (kg/m²) Operation time (min) Blood loss (mL) dFN (mm) JOA score Diagnosis Side (R/L) Crowe classification	74.6 ± 8.7 (56–87) 10 (100%) 152.3 ± 8.1 (135.7–165.0) 52.8 ± 8.9 (37.5–63.5) 22.6 ± 2.5 (18.4–26.5) 107 ± 22.7 (72–147) 248.2 ± 87.6 (125–372) 20.2 ± 2.3 (17.4–24.7) 48.1 ± 10.9 (25–61) OA:10 9/1 I:10	66.6 ± 14.5 (30–79) 8 (80%) 154.2 ± 9.1 (145.8–177.0) 61.6 ± 13.3 (41.4–86.0) 25.9 ± 5.0 (18.0–34.5) 102 ± 18.3 (78–136) 199.6 ± 50.0 (115–282) 22.4 ± 1.0 (21.0–23.9) 47.1 ± 11.9 (29–65) OA:8, ION:2 5/5 I:9, II:1	0.18 0.14 0.66 0.12 0.10 0.62 0.18 0.10 0.61 0.14 0.051 0.30

Values are presented as means and standard deviation, with the range in parentheses

BMI, body mass index; dFN, distance between the femoral nerve and the anterior acetabular edge; JOA, Japanese Orthopaedic Association; OA, osteoarthritis; ION, idiopathic osteonecrosis; R, right; L, left

Correlation coefficient: 0.66 (*p* = 0.02, moderate positive). dFN, distance between the femoral nerve and the anterior acetabular edge.

## Discussion

In this study, we compared quadricep MEP amplitudes between the supine and lateral positions during primary THA using the anterolateral approach. We observed a higher potential reduction in the supine position than in the lateral position. One strength of this study was our focus on the dFN and investigation of the differences between the supine and lateral positions.

Neurological monitoring using MEP is widely used in brain and spinal surgery. A study of 1,055 patients undergoing cervical spine surgery reported sensitivity and specificity values for EMG, SEP, and MEP of 46% and 73%, 52% and 100%, and 100% and 96%, respectively [11]. Regarding the effectiveness in THA, Shimazu et al. and Thatcher et al. reported that MEP is effective for assessing the intraoperative sciatic nerve in posterolateral THA [12, 13].

In addition to direct injury, other possible mechanisms underlying the development of nerve palsy after THA include indirect compression, traction, thermal injury due to cementation, and blood speciation. Fleischman et al. reported an overall incidence of femoral nerve palsy of 0.21%. The rate was 14.8-fold higher for anterior approaches, with 0.40% and 0.64% for direct anterior and anterolateral approaches, respectively [2]. An anterior acetabular retractor compresses the femoral nerve directly or indirectly in the anterior system approach [14, 15]. Slater et al. used a pressure transducer to measure femoral nerve pressure during THA procedures and found that only the anterior acetabular retractor placement, and not femoral head dislocation, hip adduction during femoral manipulation, or post-implant insertion repositioning, was involved in the pressure increase [14].

Short stature and low dFN may serve as risk factors for femoral nerve palsy; moreover, the supine position decreases dFN compared to the lateral position during surgery, which may be a risk factor for femoral nerve palsy [8, 9]. However, to our knowledge, no studies have reported reduced intraoperative potential of the femoral nerve innervating the muscles during THA using the modified Watson–Jones approach. Therefore, in this study, we examined the intraoperative MEP via the femoral nerve in the supine and lateral positions during retractor placement. The supine position group showed a significantly greater potential reduction in the femoral nerve compared to the lateral position group, suggesting that the supine position is a risk factor for femoral nerve palsy due to the shortening of the dFN. We also observed a moderately to strongly significant positive correlation between the dFN and the residual potential, supporting this finding. Lateral position surgery may be preferable in patients with short stature and those at high risk for femoral nerve palsy. The American Society of Neurophysiological Monitoring guidelines state that no single criteria exist for MEP amplitude reduction to fit all surgeries and that criteria should be tailored to the type of surgery [16]. In general, a residual potential amplitude of 20–50% or less is often defined as nerve damage in MEP; however, the literature reporting reduction criteria in the hip joint region is limited, and further research is needed. In this study, some cases in the supine group showed a decrease in MEPs to less than 20% of the baseline. However, no postoperative neurological deficits were observed. This may be because the duration of neural impact caused by the insertion of the retractor was short, which could have prevented permanent neurological damage. Nevertheless, previous studies have suggested that a reduction of 50–80% in MEP amplitude is indicative of nerve damage, and the findings of this study do not align with these reports. Further investigations are needed to clarify the criteria for predicting nerve injury based on MEP changes.

The limitations of this study include its small sample size. Additionally, this study evaluated the potentials during THA surgery rather than paralysis itself. However, potential reduction is reportedly associated with femoral nerve palsy. Moreover, the lack of control MEP recordings on the non-operative side prevents us from fully addressing concerns related to time-dependent variability in MEP. Furthermore, the use of muscle relaxants during anesthesia induction may have affected the potential. Although TOF values were measured, the absence of clearly defined criteria for evaluating the degree of neuromuscular blockade may have affected the MEP waveforms. However, the method of anesthesia did not change because of differences in surgical position during the study period.

## Conclusion

For primary THA using the anterolateral approach, quadriceps MEP amplitude reduction occurred during acetabular retractor placement and was significantly larger in the supine position than in the lateral position. Therefore, the lateral position may decrease the risk of femoral nerve palsy after THA compared to the supine position. Future studies with larger sample sizes are needed to evaluate the effects of muscle relaxants and other types of anesthesia on outcomes; similarly, methods that more directly assess paralysis may help validate the findings of the present study.

## Data Availability

The data that support the findings of this study are available from the corresponding author upon reasonable request.
